# Epidermoid Cyst of the Gingiva: A Rare Case Report and Literature Review

**DOI:** 10.1002/ccr3.72671

**Published:** 2026-05-13

**Authors:** José Alcides Almeida de Arruda, Sicília Rezende Oliveira, Marcela Ferreira Abrahão Ribeiro, Martinho Campolina Rebello Horta, Giovanna Ribeiro Souto

**Affiliations:** ^1^ Graduate Program in Dentistry Pontifícia Universidade Católica de Minas Gerais Belo Horizonte Brazil; ^2^ School of Dentistry Universidade Federal de Alfenas Alfenas Brazil; ^3^ Department of Oral Surgery, Pathology, and Clinical Dentistry, School of Dentistry Universidade Federal de Minas Gerais Belo Horizonte Brazil

**Keywords:** differential diagnosis, epidermal cyst, gingiva, oral diagnosis, oral medicine

## Abstract

Oral epidermoid cysts are developmental lesions that account for less than 0.04% of all reported cases and typically occur in the floor of the mouth; gingival involvement is exceedingly rare. This report describes a 23‐year‐old female presenting with an asymptomatic, whitish papule on the attached gingiva near the right mandibular canine. Histopathologically, the lesion showed a cystic cavity mainly lined by orthokeratinized stratified squamous epithelium containing lamellar keratin, without cutaneous adnexal structures in the fibrous connective tissue capsule. No recurrence was observed after 15 months of follow‐up. A literature review identified eight epidermoid cysts involving the gingiva or alveolar ridge. This report underscores the importance of including this entity in the differential diagnosis of gingival papules and indicates that accurate clinical recognition coupled with histopathological confirmation ensures curative surgical outcomes.

## Key Clinical Message

Gingival epidermoid cyst may clinically resemble common reactive or benign proliferative lesions, leading to diagnostic uncertainty. Recognition of this rare topographic presentation and mandatory histopathological confirmation are essential to establish the correct diagnosis and ensure curative surgical management with excellent prognosis.

## 1 Introduction

Epidermoid cysts are benign lesions of ectodermal origin that are rarely found in the oral cavity. They represent fewer than 0.01% of all oral cysts and approximately 1.6% of cases occurring in the head and neck region [1, 2, 3, 4]. In Brazil, studies indicate that epidermoid cysts account for 0.07% to 0.08% of oral and maxillofacial biopsies [4, 5].

Epidermoid, dermoid, and teratoid cysts are classically grouped under the designation of dysontogenic cysts, a classification based on the presence of cutaneous adnexal structures and elements derived from different embryonic germ layers [6, 7, 8]. The pathogenesis of epidermoid cysts remains partially understood, with both congenital and acquired origins related to trauma having been proposed [2, 4]. Histopathologically, these cysts are lined by orthokeratinized stratified squamous epithelium supported by a thin fibrous connective tissue capsule, without evidence of cutaneous adnexa such as hair follicles or sebaceous glands, and their lumen contains concentric lamellae of eosinophilic keratin [8].

Clinically, epidermoid cysts present as well‐circumscribed, slow‐growing, painless nodules covered by normally colored mucosa. They most commonly occur in middle‐aged adults and show a slight male predominance [5]. The lip and the floor of the mouth are the most frequently affected sites, accounting for 31.7% and 30.7% of cases, respectively [4, 5]. Gingival involvement, however, is exceptionally rare, with only a few cases reported in the literature [4, 5, 9]. We herein report an additional case of a gingival epidermoid cyst and provide a brief review of all previously documented cases.

## 2 Case Report

### 2.1 Case History

A 23‐year‐old Brazilian woman was referred to the Oral Medicine service of the Department of Dentistry at the Pontifícia Universidade Católica deMinasGerais (PUC Minas) for evaluation of a small gingival lesion of approximately 1 year's duration, which had shown slow, progressive growth without associated symptoms. No history of alcohol or tobacco use was reported, nor were any relevant comorbidities or contributory systemic conditions identified. The patient was on daily oral contraceptive therapy and denied parafunctional habits or episodes of local trauma.

**FIGURE 1:**
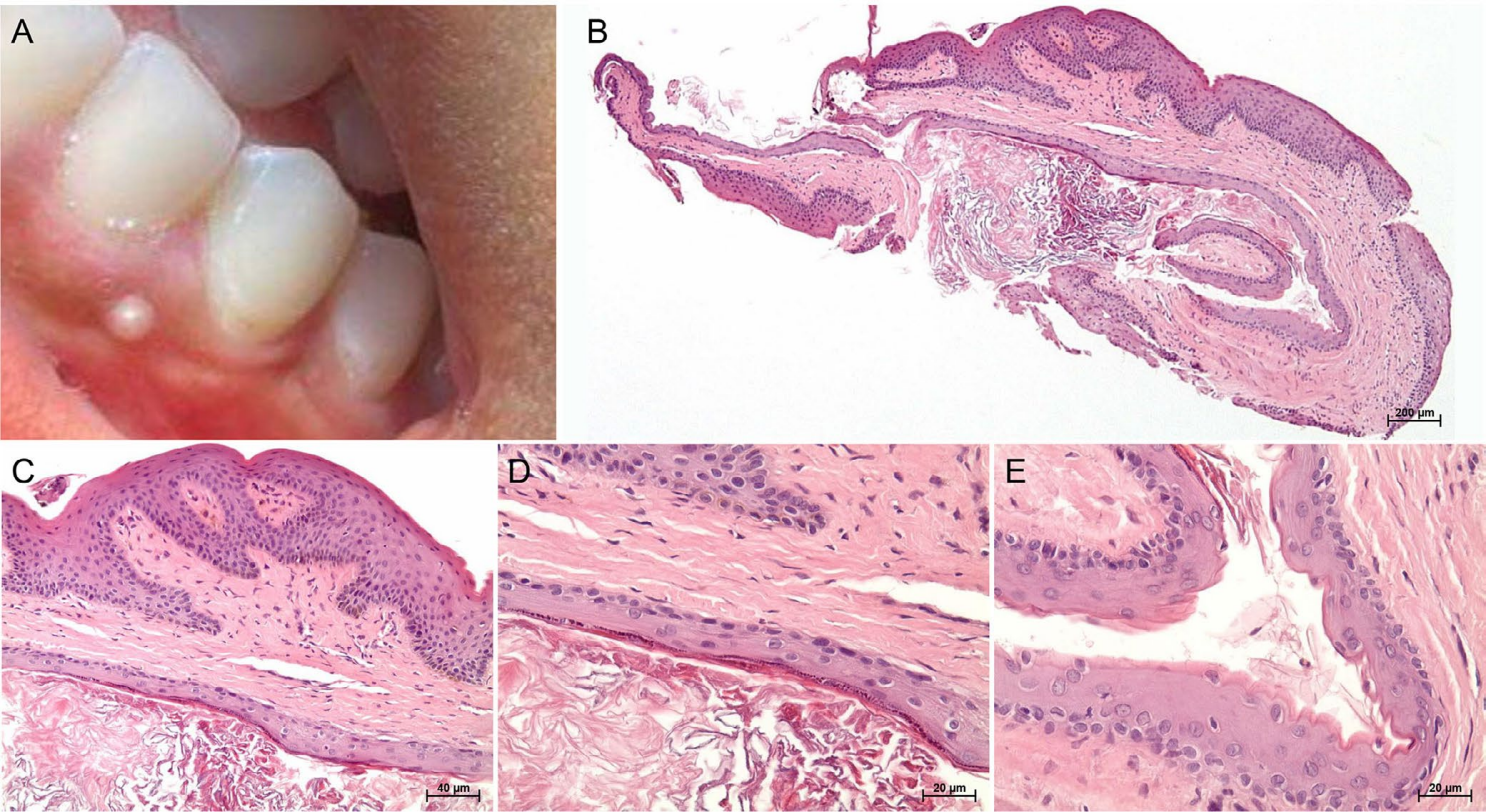
Clinical and histopathological features of the epidermoid cyst. (A) Intraoral view showing a solitary, well‐circumscribed, whitish papule located on the attached gingiva adjacent to the right mandibular canine, measuring approximately 2 × 2 mm. The lesion had a smooth surface, rounded contour, and sessile base (horizontally flipped image, as it is a patient‑taken selfie). (B) Low‐power photomicrograph showing a cystic cavity beneath the lamina propria of the oral mucosa. (C) The cyst is mainly lined by orthokeratinized stratified squamous epithelium surrounded by a thin fibrous capsule without adnexal structures. (D) Higher magnification of the orthokeratinized stratified squamous epithelium with a well‐developed granular layer. (E) Focal area of undulated parakeratinized surface in the cyst lining epithelium (hematoxylin and eosin; magnification: B, ×40; C, ×200; D and E, ×400).

Intraoral examination revealed a solitary, firm, well‐circumscribed whitish papule measuring 2 × 2 mm on the attached gingiva adjacent to the right mandibular canine (Figure 1A).

### 2.2 Differential Diagnosis

Based on the clinical presentation, the diagnostic hypotheses considered included verruca vulgaris, adult gingival cyst, and focal fibrous hyperplasia.

### 2.3 Treatment and Investigation

The lesion was completely excised under local anesthesia. Gross examination revealed a small, firm, whitish soft‐tissue fragment. Histopathological analysis demonstrated a cystic cavity predominantly lined by orthokeratinized stratified squamous epithelium with a well‐developed granular layer and lamellar keratin within the lumen, and a thin fibrous connective tissue capsule without cutaneous adnexal structures (Figure 1B–E). These features supported the histopathological diagnosis of an epidermoid cyst.

### 2.4 Outcome and Follow‐Up

No recurrence was observed after 15 months of follow‐up.

## 3 Discussion

Data obtained from the literature review confirm that gingival/alveolar ridge epidermoid cysts represent an extraordinarily rare presentation, with only eight previously documented cases (Table 1). Publications are concentrated in Brazil and India [4, 5, 9, 10]. Overall, a male predominance was observed (62.5%), with a mean age of 35.3 years and an average clinical duration of 3.2 years. By contrast, our patient was a 23‐year‐old woman with an estimated one‐year clinical history, placing her at the younger end of the spectrum and highlighting the demographic heterogeneity of this atypical presentation.

**TABLE 1:** Summary of reported cases of epidermoid cysts in the gingiva/alveolar ridge in the literature^a^.

Study	Number of cases	Sex	Age	Time of evolution	Clinical features	Treatment	Outcome
Santos et al., 2020 [4]	2	M	11 months	NI	Asymptomatic, pedunculated, oval‐shaped lesion, soft in consistency, yellowish in color, measuring 0.8 × 0.8 cm, located on the lingual gingival surface near the right maxillary second premolar	Surgical excision	NI
		F	46 years	NI	Sessile, nodular lesion with slow growth, firm consistency, yellowish color, measuring 2 × 1 cm, located on the alveolar ridge near the buccal region	NI	NI
Cunha et al., 2023 [5]	2	NI	NI	NI	One case involving the gingiva and another the alveolar ridge	Surgical excision	NI
Ravindranath et al., 2009 [9]	3	M	70 years	7 years	Asymptomatic swelling in the posterior gingival region of the right mandibular molars	Surgical excision	No recurrence
		M	22 years	1 year	Asymptomatic swelling with slow growth in the posterior gingival region of the left mandibular molars	Surgical excision	No recurrence
		M	45 years	4 years	Asymptomatic swelling in the posterior gingival region of the left mandibular molars	Surgical excision	No recurrence
Puranik et al., 2016 [10]	1	M	28 years	1 year	Single, well‐defined, oval‐shaped, asymptomatic swelling (~3.5 × 2.5 cm) on the lingual posterior gingiva of the left mandibular second premolar and first and second molars	Surgical excision	1 year; no recurrence

Abbreviations: M, male; NI, not informed.

^a^ Searches were conducted without restrictions in PubMed, Web of Science, Scopus, Embase, LILACS, and Google Scholar in September 2025. The following search strategy was used: (“epidermoid cyst” OR “developmental cyst” OR “dysontogenic cyst”) AND (“alveolar ridge” OR “mouth mucosa” OR “oral cavity” OR “oral mucosa” OR “oral mucosae” OR gingiva OR gingivae OR mouth). A total of 914 records were retrieved from the electronic databases (PubMed: 176; Web of Science: 131; Scopus: 415; Embase: 171; LILACS: 21), of which four articles comprising eight cases were included in the qualitative analysis.

Clinically, the revisited cases mostly described firm, asymptomatic, slowly enlarging masses ranging from 0.8 to 3.5 cm [4, 9, 10]. The present case, however, differed by its minute size (2 mm) and whitish papular morphology, i.e., features that supported the initial clinical hypotheses of verruca vulgaris, adult gingival cyst, and focal fibrous hyperplasia. This clinical resemblance to non‐neoplastic proliferative processes reinforces the importance of histopathological examination for any gingival lesion, even when asymptomatic [11, 12]. In this regard, a clinicopathologic discrepancy has been reported in 75.9% of oral epidermoid cysts, underscoring the risk of diagnostic error when microscopic confirmation is omitted [5].

Epithelial lesions and gingival cysts comprise a spectrum of benign conditions that may share overlapping clinical features. Focal fibrous hyperplasia, for instance, is a common hyperplastic reaction generally associated with chronic trauma (e.g., prosthetic irritation or parafunctional habits), occurring predominantly in women between the second and fifth decades of life [4]. Histopathologically, it presents as dense fibrous stroma covered by hyperplastic squamous epithelium, sometimes with hyperkeratosis or superficial ulceration. Verruca vulgaris, in turn, is induced by human papillomaviruses (HPV) and typically appears as a papillary papule or nodule, most often in children and adolescents; microscopically, it shows acanthosis, papillomatosis, and koilocytosis [13]. Although these conditions fall within the clinical differential diagnosis of benign gingival lesions, the histological pattern observed in the present case excludes reactive or infectious etiologies. Among odontogenic cysts, the adult gingival cyst is noteworthy, representing approximately 0.3% of odontogenic cysts. It typically affects the gingiva of middle‐aged women and presents as a raised lesion, sometimes whitish, with an average size of 5.8 mm. Histopathologically, it is characterized by a cystic cavity lined by thin, nonkeratinized stratified epithelium [14, 15].

The histopathological features observed in the present case, i.e., orthokeratinized and parakeratinized stratified squamous epithelium, lamellar keratin within the lumen, and absence of cutaneous adnexa in the cystic capsule, align with the classic phenotype of epidermoid cysts, clearly distinguishing them from dermoid cysts [4, 5, 7, 8]. As observed in the reported case, focal areas of parakeratinization may occasionally be seen in oral epidermoid cysts [4]. The presence of a cystic cavity lined by stratified squamous epithelium and the absence of cutaneous adnexal structures in the fibrous capsule are considered relevant diagnostic criteria [8]. Within the spectrum of dysontogenic cysts, their pathogenesis may result from congenital ectodermal entrapment during embryonic fusion or from acquired implantation of surface epithelium secondary to trauma [2, 8]. In the present case, the lack of features suggestive of congenital origin and the clinical duration support an acquired lesion, consistent with superficial epithelial implantation mechanisms described for oral epidermoid cysts. It is also important to note that, from a differential standpoint, teratoid cysts (also included within the dysontogenic group) are distinguished by their congenital nature, predominance in the first decade of life, and sublingual location. Histopathologically, they may exhibit squamous, respiratory, or gastrointestinal epithelium, along with mesodermal/endodermal derivatives such as glands, cartilage, or bone [8, 16].

Complete surgical excision is the treatment of choice for epidermoid cysts, a strategy consistently employed across nearly all reported cases, with no recurrence observed when complete removal is achieved [4, 5, 9, 10]. The outcome in our patient, i.e., 15 months of recurrence‐free follow‐up, is entirely consistent with this pattern, reinforcing the excellent prognosis and curative nature of the lesion when adequately excised. Recurrences, although rare, have usually been associated with partial removal, particularly in lesions located in the floor of the mouth [17].

As a case report, this study has inherent limitations and does not allow population‐level inferences. Nevertheless, it contributes to the literature by documenting an exceptionally rare presentation [18]. Notably, large retrospective studies on gingival lesions, including those by Alblowi and Binmadi [11] (119 cases over 20 years) and Montazer Lotf‐Elahi et al. [12] (1000 cases over 22 years), did not identify any epidermoid cysts. Therefore, the relevance of the present report lies in both its rarity and the comprehensive review of previously published cases.

In summary, this ninth documented case of a gingival epidermoid cyst reinforces that this topographic presentation may pose a diagnostic challenge—even in young adults—countering traditional heuristics that associate the lesion with the floor of the mouth and older age groups. Two key messages emerge: (i) epidermoid cysts should be included in the differential diagnosis of firm, whitish gingival papules or nodules, alongside non‐neoplastic proliferative processes, benign epithelial lesions, and other peripheral (extraosseous) odontogenic cysts or tumors; and (ii) histopathological confirmation is essential, as complete excision is typically curative and the prognosis is excellent.

## Author Contributions

**José Alcides Almeida de Arruda:** conceptualization, data curation, formal analysis, investigation, methodology, writing – original draft, writing – review and editing. **Sicília Rezende Oliveira:** conceptualization, formal analysis, methodology, writing – original draft, writing – review and editing. **Marcela Ferreira Abrahão Ribeiro:** conceptualization, data curation, methodology, project administration, visualization, writing – review and editing. **Martinho Campolina Rebello Horta:** conceptualization, investigation, methodology, project administration, writing – original draft. **Giovanna Ribeiro Souto:** conceptualization, formal analysis, methodology, supervision, writing – original draft, writing – review and editing.

## Funding

This study was financed in part by the Coordenação de Aperfeiçoamento de Pessoalde Nível Superior – Brasil (CAPES) – Finance Code 001. S.R.O. is a research fellow supported by the Conselho Nacional de Desenvolvimento Científico e Tecnológico (CNPq) (150011/2024‐5).

## Consent

Data were collected in accordance with the guidelines of the institutional Research Ethics Board. Written informed consent was obtained from the patient for data collection and publication of this case report and accompanying images.

## Conflicts of Interest

The authors declare no conflicts of interest.

## Data Availability

Research data are not shared.
